# Surgical strategy for polydactyly of the thumb

**DOI:** 10.1186/1753-6561-9-S3-A10

**Published:** 2015-05-19

**Authors:** Goo Hyun Baek

**Affiliations:** 1 Department of Orthopaedic Surgery, Seoul National University Hospital, Seoul, 110-744, Korea

## 

Polydactyly of the thumb, radial polydactyly, or preaxial polydactyly is common in all races, and about 20 percent of them occur bilaterally. Patients with polydactyly of the thumb show very diverse manifestations, from a rudimentary floating type to a complex one.

The goal of surgical reconstruction is to make a straight, mobile, and stable thumb of good appearance in size and shape. The simple ablation of one digit has not produced satisfactory outcomes in most cases of polydactyly of the thumb, and resulted in retained deviation, stiffness and/or ligamentous instability of the thumb. Surgical concepts and techniques are still evolving. However, there are several reconstructive strategies to achieve a functionally and cosmetically acceptable thumb.

## Excision

### Indication

When an accessory thumb is attached only by a small soft tissue pedicle, or when there is no bony connection between two thumbs, simple excision of minor thumb is indicated. The dominant thumb should have good stability, motion and appearance.

## Reconstruction

### Indication

One thumb is well developed, and the other one is less developed.

There might be bony or cartilaginous connection between two thumbs (Wassel types I, III, V), or two thumbs share a joint (Wassel types II, IV, VI, VII).

### Technique

**Figure 1:**
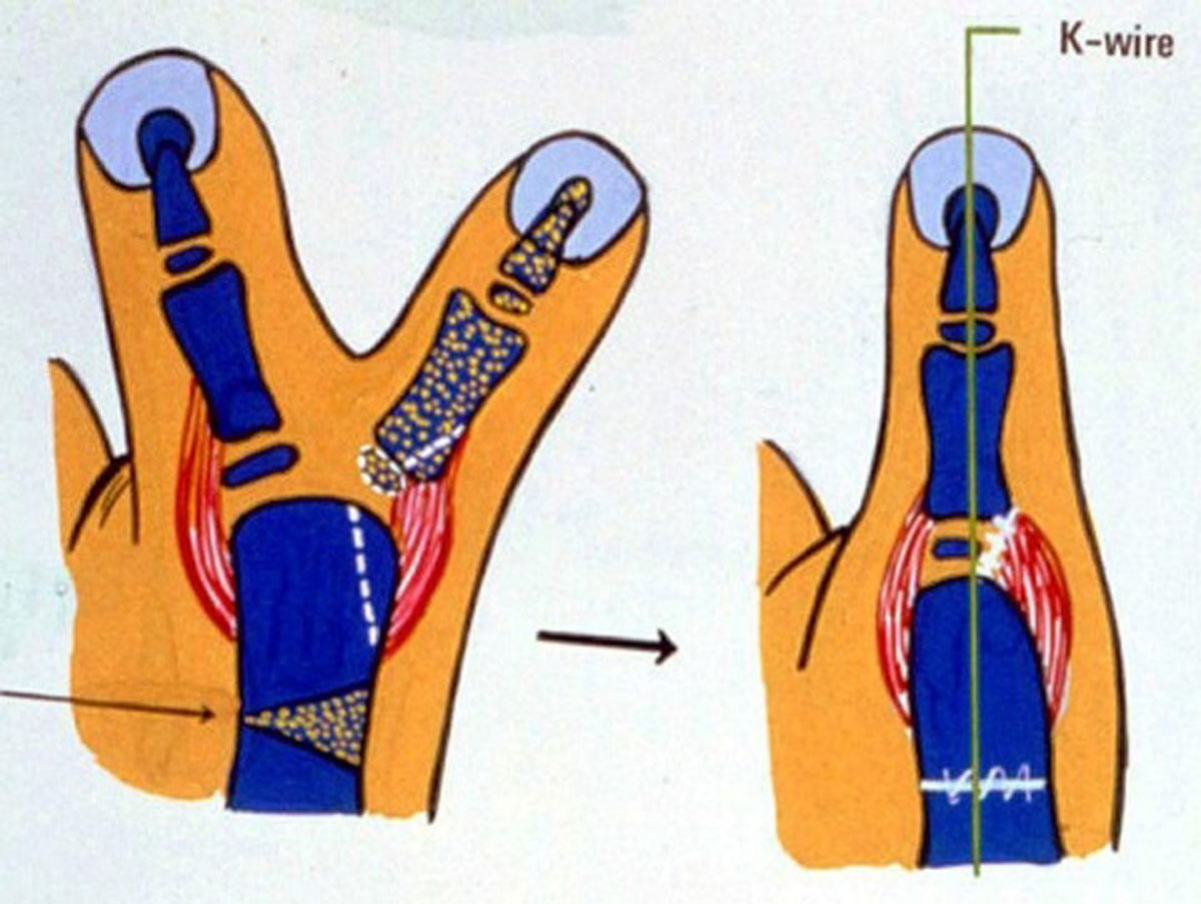
Ligamentoperiosteal flap was raised to reconstruct radial collateral ligament of MP joint. Metacarpal head was excised partially to fit base of dominant proximal phalanx. Corrective osteotomy was added to make a straight thumb

Reconstruction procedure includes arthroplasty, corrective osteotomy, and tendon realignment (Figure 1). During arthroplasty of metacarpophalangeal or interphalangeal joint, elevation of ligamentperiosteal flap is very important to reconstruct stable collateral ligament. When metacarpal or proximal phalangeal head is too big to fit the above phalangeal bone, partial head excision is necessary to make a proper joint. Angular deformity can be corrected by corrective wedge osteotomy. When there are abnormal insertions of FPL and/or EPL tendons, the insertion sites should be realigned to achieve good flexion-extension arc.

## Combination procedure (modified Bilhaut-Cloquet procedure)

### Indication

Both thumbs are hypoplastic and show almost symmetric appearance.

Especially when the nail width is less than 2/3 of contralateral normal side in unilateral cases, and when the nail width is less than that of index finger in bilateral cases.

## On-top plasty

### Indication

One thumb has well developed proximal part and poorly developed distal part (nails are frequently absent) *at the phalangeal level*, on the other hand, the other thumb has a poorly developed proximal part and a well developed distal part.

## Ray transfer

### Indication

One thumb has a well developed proximal part and a poorly developed distal part *at metacarpal level*, and on the other hand, the other thumb has a poorly developed proximal part of metacarpal and a well developed distal part.

## Atypical cases

For atypical types of polydactyly of the thumb such as triplicated thumb, surgical plan should be considered individually. Some of the techniques described above can be combined, and additional procedures like pollicization is needed to produce a good thumb.
